# The consensus rye microsatellite map with EST-SSRs transferred from wheat

**DOI:** 10.18699/VJ20.48-o

**Published:** 2020-08

**Authors:** D.O. Vidakovic, D. Perovic, T.V. Semilet, A. Börner, E.K. Khlestkina

**Affiliations:** Julius Kuehn-Institute (JKI), Quedlinburg, Germany University of Novi Sad, Department of Biology and Ecology, Novi Sad, Serbia; Julius Kuehn-Institute (JKI), Quedlinburg, Germany; Federal Research Center the N.I. Vavilov All-Russian Institute of Plant Genetic Resources (VIR), St. Petersburg, Russia; Leibniz Institute of Plant Genetics and Crop Plant Research (IPK), Gatersleben, Germany; Federal Research Center the N.I. Vavilov All-Russian Institute of Plant Genetic Resources (VIR), St. Petersburg, Russia Institute of Cytology and Genetics of Siberian Branch of the Russian Academy of Sciences, Novosibirsk, Russia Novosibirsk State University, Novosibirsk, Russia

**Keywords:** Secale cereale, SSR, Triticum aestivum, microsatellite markers, genetic mapping, Secale cereale, SSR, Triticum aestivum, микросателлитные маркеры, генетические карты

## Abstract

Microsatellite (SSR) markers with known precise intrachromosomal locations are widely used for mapping genes in rye and for the investigation of wheat-rye translocation lines and triticale highly demanded for mapping economically important genes and QTL-analysis. One of the sources of novel SSR markers in rye are microsatellites transferable from the wheat genome. Broadening the list of available SSRs in rye mapped to chromosomes is still needed, since some rye chromosome maps still have just a few microsatellite loci mapped. The goal of the current study was to integrate wheat EST-SSRs into the existing rye genetic maps and to construct a consensus rye microsatellite map. Four rye mapping populations (P87/P105, N6/N2, N7/N2 and N7/N6) were tested with CFE (EST-SSRs) primers. A total of 23 Xcfe loci were mapped on rye chromosomes: Xcfe023, -136 and -266 on chromosome 1R, Xcfe006, -067, -175 and -187 on 2R, Xcfe029 and -282 on 3R, Xcfe004, -100, -152, -224 and -260 on 4R, Xcfe037, -208 and -270 on 5R, Xcfe124, -159 and -277 on 6R, Xcfe010, -143 and -228 on 7R. With the exception of Xcfe159 and Xcfe224, all the Xcfe loci mapped were found in orthologous positions considering multiple evolutionary translocations in the rye genome relative to those of common wheat. The consensus map was constructed using mapping data from the four bi-parental populations. It contains a total of 123 microsatellites, 12 SNPs, 118 RFLPs and 2 isozyme loci.

## Abstract (ru)

Микросателлитные (SSR) маркеры широко используют для картирования генов ржи и анали- за транслокационных линий пшеницы и тритикале. SSR-маркеры с известной внутрихромосомной локализацией очень востребованы для картирования экономически значимых генов и QTL- анализа. Одним из источников новых SSR-маркеров у ржи являются микросателлитные маркеры пшеницы. Несмотря на не- сколько наборов микросателлитных маркеров, доступных у ржи, по-прежнему необходимо расширение списка SSR, сопоставленных с хромосомами ржи, поскольку на некоторых генетических картах количество SSR-маркеров невелико. Цель настоящего исследования состояла в том, чтобы интегрировать EST-SSR пшеницы в существующие генетические карты ржи и построить консенсусную микросателлитную карту ржи. Четыре картирующих популяции ржи (P87/P105, N6/N2, N7/N2 и N7/N6) тестировали с использова- нием праймеров CFE (EST-SSR). В результате в молекулярно-генетические карты ржи было интегрирова- но 23 микросателлитных локуса Xcfe: Xcfe023, -136 и -266 на хромосоме 1R, Xcfe006, -067, -175 и -187 на 2R, Xcfe029 и -282 на 3R, Xcfe004, -100, -152, -224 и -260 на 4R, Xcfe037, -208 и -270 на 5R, Xcfe124, -159 и -277 на 6R, Xcfe010, -143 и -228 на 7R. За исключением Xcfe159 и Xcfe224, все картированные локусы Xcfe были обнаружены в ортологичных позициях с учетом множественных транслокаций в ходе эволюции генома ржи по сравнению с пшеницей. Консенсусная карта построена с использованием данных по четырем картирую- щим популяциям ржи. Она содержит в общей сложности 123 микросателлитных маркера, 12 SNP, 118 RFLP и 2 изоферментных локуса

## Introduction

Several linkage maps of rye carrying RFLP, AFLP, SSR, DArT and SNP markers are available to date (Devos et al., 1993; Philipp et al., 1994; Senft, Wricke, 1996; Korzun et al., 2001; Bednarek et al., 2003; Hackauf, Wehling, 2003; Khlestkina et al., 2004; Varshney et al., 2007; Bolibok-Brągoszewska et al., 2009; Gustafson et al., 2009; Milczarski et al., 2011, 2016; Xu et al., 2012; Bauer et al., 2017).

SSRs (microsatellites) are among the most widely used DNA-markers in rye genetics. For example, SSR markers were used for mapping the sy1, sy9, sy18 and sy19 asynaptic genes (Malyshev et al., 2009; Dolmatovich et al., 2013a, b), the gene mo1 for supernumerary spikelets (Dobrovolskaya et al., 2009), several anthocyanin biosynthesis genes (Khlestkina et al., 2009, 2011, 2013), Ddw1 (Tenhola-Roininen, Tanhuanpää, 2010) and Ddw3 (Yang et al., 2018) dwarfing genes, the powdery mildew resistance locus (Wang et al., 2010), the Elm-R1 gene related with embryo lethality in wheat-rye hybrids as well as the hybrid dwarfness gene Hdw-R1 (Tikhenko et al., 2011; Tsvetkova et al., 2018), aluminum tolerance loci in rye and triticale (Fontecha et al., 2007; Benito et al., 2010; Niedziela et al., 2014) and several QTL for agronomic traits including grain yield (Hackauf et al., 2017).

SSRs can be suitable for marker-assisted breeding (Lapitan et al., 2007), detection of the genetic variability in rye and triticale (Bolibok et al., 2005; Vyhnánek et al., 2009) as well as for marker-assisted identification of rye genetic material in wheat cultivars and lines (Silkova et al., 2006; Schlegel, Korzun, 2008; Schneider, Molnár-Láng, 2009; Adonina et al., 2011; Silkova et al., 2011; Schlegel, 2015).

In spite of several sets of microsatellite markers available in rye, broadening a list of SSRs mapped to rye chromosomes is still needed, since some rye chromosome maps still have just a few microsatellite loci mapped (Khlestkina et al., 2004). The goal of the current study was to integrate wheat EST-SSRs, expressed sequence tag SSR (from map of L.Y. Zhang et al. (2005)) into the existing rye microsatellite map and construct the consensus microsatellite map of rye genome.

## Materials and methods

Four rye F_2_ mapping populations (P87/P105; N6/N2, N7/N2 and N7/N6; see detailes in (Khlestkina et al., 2004)) were used in PCR assays with CFE primers available at GrainGenes database (http://wheat.pw.usda.gov). DNA was available from previous studies (Korzun et al., 2001; Khlestkina et al., 2004). PCR and analysis of the amplified fragments length was performed as described in L.Y. Zhang et al. (2005). Chromosome arm location of homologous sequences carrying the CFEs (http://wheat.pw.usda.gov) was performed using BLAST analysis (Altschul et al., 1990) of the corresponding wheat ESTs given at http://wheat.pw.usda.gov against wheat chromosome survey sequences available at https://urgi.versailles.inra.fr/blast/blast.php. Linkage maps were constructed with MAPMAKER 2.0 (Lander et al., 1987) using Kosambi function (Kosambi, 1944), based on genotyping data obtained in the current study and previously (Korzun et al., 2001; Khlestkina et al., 2004; Varshney et al., 2007). The consensus map was constructed using JoinMap 2.0 program (Stam, 1993).

## Results and discussion

Despite the possibility of a high‐throughput marker analysis using SNPs (Bauer et al., 2017), microsatellites remain convenient and low‐cost markers for mapping genes and marker assisted selection in rye and triticale. For these purposes microsatellite markers with known precise intrachromosomal location are needed. The sources for mapping novel SSR loci in rye were rye EST-SSRs (Hackauf, Wehling, 2003; Khlestkina et al., 2004), or wheat genomic microsatellites (Khlestkina et al., 2004). In the current study, we used wheat EST-SSRs for genotyping rye mapping populations.

The parents of the four rye mapping populations (P87/P105, N6/N2, N7/N2 and N7/N6) were tested with 301 CFE primer pairs. Thirty-two pairs revealed polymorphism between the parents of one or more mapping populations: 10 between P87 and P105, 13 between N6 and N2, 11 between N7 and N2 and 15 between N7 and N6. The portion of polymorphic CFE markers (10.6 %) is comparable with that described for genomic wheat SSRs GWM transferred to the same set of mapping populations parents (9.2 %) (Khlestkina et al., 2004).

Twenty-three of the 32 markers were segregating in the mapping populations, while nine pairs produced monomorphic PCR-products, that can be explained by rye heterogeneity. Twenty-three Xcfe loci were genetically mapped on rye chromosomes (see the Table and Supplementary Materials)^1^.


^1^Supplementary Materials are available in the online version of the paper: http:/www.bionet.nsc.ru/vogis/download/pict-2020-24/appx5.pdf



A consensus map was constructed using mapping data for the four populations. The consensus map contains 11 microsatellite (Xcfe…, Xrems… or Xgwm…) markers on chromosome 1R, 23 on 2R, 10 on 3R, 15 on 4R, 29 on 5R, 17 on 6R, 18 on 7R (see the Figure). In addition to these 123 SSR markers the consensus map contains 12 SNPs (Xgbs…), 118 RFLP markers (other X… names), and two isozyme loci. The former rye consensus map constructed in 2009 contained 10 microsatellite markers only (Gustafson et al., 2009).

Most of the microsatellites mapped in the current study consist of 3 bp repeats (15 loci), 5 of the mapped SSRs were dinucleotide, 2 sequences carried tetra- and 1 hexanucleotide repeat (see the Table).

**Table 1.**
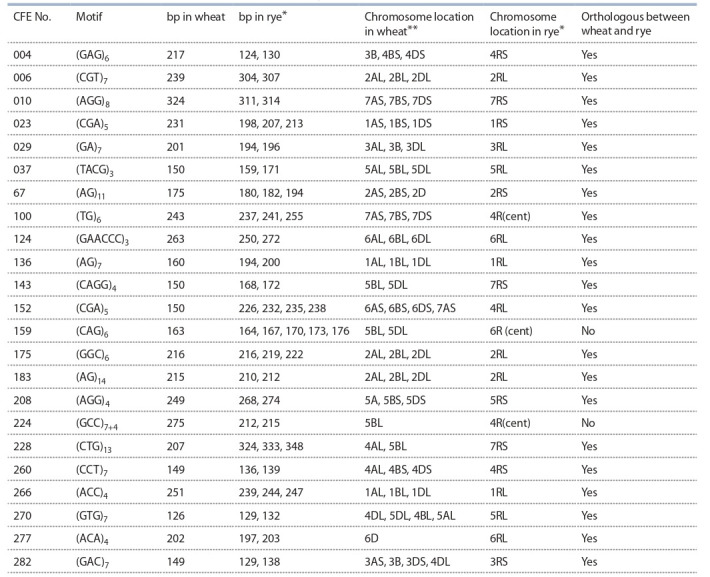
Characterization of CFE markers mapped in the current study * Data obtained in the current study (different length of the PCR products correspond to different parents of the rye mapping populations used; each microsatellite studied was monolocus and homozygous in all parents of the mapping populations, amplifying one fragment in each parental genotype). ** Chromosome location of homologous sequences carrying the CFEs (http://wheat.pw.usda.gov) was performed using BLAST analysis of the corresponding wheat ESTs given at http://wheat.pw.usda.gov against wheat chromosome survey sequences available at https://urgi.versailles.inra.fr/blast/blast.php. Further information is given according to http://wheat.pw.usda.gov.

Twenty-one of the 23 Xcfe loci mapped in orthologous positions (see the Table) considering multiple evolutionary translocations in the rye genome relative to those of common wheat, as described in detail by K.M. Devos et al. (1993). Two loci Xcfe159-6R and Xcfe224-4R have no orthology with wheat Xcfe159 (5A, 5D) and Xcfe224 (5B). The portion of the Xcfe loci showing orthology between wheat and rye (91 %) is higher than that found previously for genomic SSR loci Xgwm (73 %) (Khlestkina et al., 2004). This may reflect conservatism of the coding portion of plant genome, in particular that of the regions complementary to the primers, flanking microsatellites.

Usually the markers mapped to 7RS are found in a comprehensive region of the chromosome 7R corresponding to ancient translocation, while just a few markers are available for the small proximal region not involved in this translocation (Devos et al., 1993; Korzun et al., 2001; Khlestkina et al., 2004). The Xcfe010-7R locus mapped in the current study is located in this region (see the Figure) and can be used for tagging the part of chromosome 7RS, which is orthologous to the short arm of chromosome 7 of Triticeae (Devos et al., 1993).

**Fig. 1.**
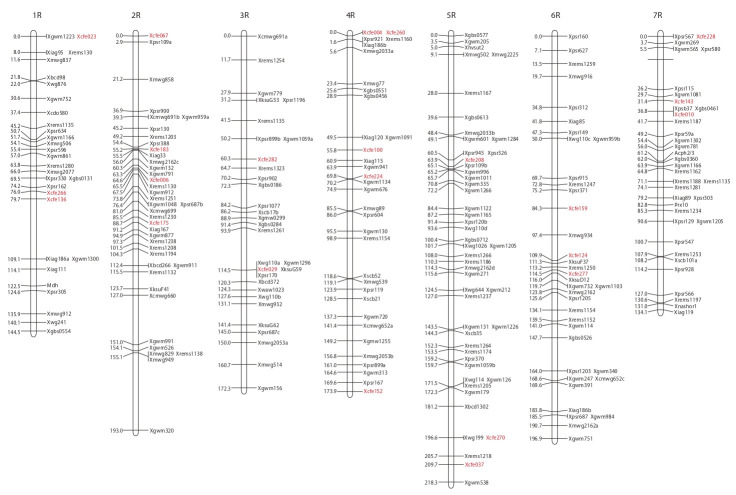
Rye consensus microsatellite map constructed in the current study based on the map data from our study (for wheat EST-SSRs) and previous studies (for SNPs, Varshney et al., 2007; rye SSRs and wheat gSSRs, Khlestkina et al., 2004; RFLPs, Korzun et al., 2001) using JoinMap 2.0 program (Stam, 1993).

The Xcfe loci mapped can be recommended for various applications in rye genetics and breeding. Some of them locate in the regions carrying known rye genes and therefore have a potential for marker-assisted selection. For example, comparison of the consensus map (see the Figure) with data available from previous gene mapping studies suggests Xcfe270-5R to be close to the dwarfing gene Ddw1 (mapped by (Tenhola- Roininen, Tanhuanpää, 2010)), while the Xcfe006-2R locus (see the Figure) is mapped in the region highly comparable with location of the asynaptic genes sy9 and sy18 on chromosome 2R (Malyshev et al., 2009; Dolmatovich et al., 2013a).

Overall, the consensus map of rye contains 123 microsatellites. The list of mapped SSRs can be broaden in the future based on 856 SSRs recently found in rye genome shotgun survey sequences (Li et al., 2018).

## Conclusion

The consensus map constructed in the current study contains a total of 123 microsatellites (including 23 SSRs transferred in our study from wheat to rye map), 12 SNPs, 118 RFLPs and 2 isozyme loci. Co-linearity between rye and wheat chromosome regions carrying these microsatellite loci was shown using 21 from 23 SSRs. These markers can be useful for both comparative mapping between wheat, rye and triticale as well as for marker-assisted breeding.

## Conflict of interest

The authors declare no conflict of interest.
